# Alternative access in transcatheter aortic valve replacement—an updated focused review

**DOI:** 10.3389/fcvm.2024.1437626

**Published:** 2024-08-08

**Authors:** Mark W. Abdelnour, Vishal Patel, Pranav M. Patel, A. M. Kasel, Antonio H. Frangieh

**Affiliations:** ^1^ Division of Cardiology, Department of Medicine, University of California Irvine Medical Center, Orange, California, CA, United States; ^2^ Department of Cardiology, University Heart Center, University Hospital Zurich and University of Zurich, Zurich, Switzerland

**Keywords:** TAVR, TAVR - current and future, TAVI, TAVR alternative access, aortic stenosis, TAVR history, transcaval access

## Abstract

Aortic Stenosis (AS) is a common condition with an estimated pooled prevalence of all AS in the elderly population at around 12.4%, with that of severe AS estimated to be around 3.4%. In the past, surgical aortic valve replacement was the primary treatment option for severe AS for decades. However, with the compelling evidence on the safety and efficacy of transcatheter aortic valve replacement (TAVR), it has become the gold standard treatment option for many patients with symptomatic severe AS. Transfemoral access has been the preferred method for transcatheter heart valve delivery. However, the prevalent use of TAVR on a diverse patient profile with different risk factors, such as peripheral artery disease, precluded the possibility of a transfemoral approach despite the improvement of valves and delivery systems technology. Therefore, alternative TAVR approaches have gained increasing utility in cases where transfemoral access is unfavorable. We review the journey, evolution, and techniques for different approaches of percutaneous TAVR, including transfemoral, transcarotid, transsubclavian/transaxillary, and transcaval approaches, in addition to the traditional “surgical” transaortic and transapical accesses. Consolidating these data highlights each approach's practicality and limitations, providing additional grounding for case-by-case utilization and future clinical research.

## Graphical Abstract

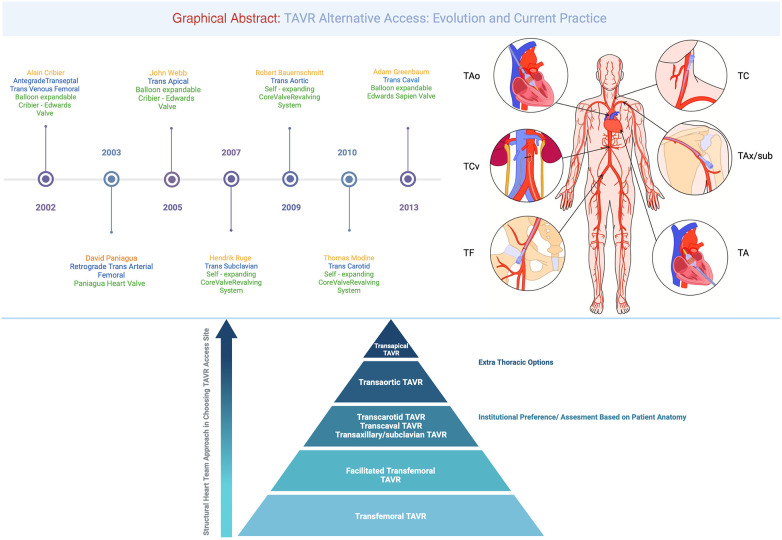

## Introduction

Aortic stenosis (AS) is an insidious disease characterized by a long latency period, followed by rapid progression after the appearance of symptoms, resulting in a high mortality rate among untreated patients (1). Although the etiology of AS can vary, cases due to calcific degeneration predominantly affect those above the age of 65. The pooled prevalence of all AS in the elderly (75 years or older) is 12.4%, with that of severe AS estimated to be around 3.4%. Moreover, the number of patients with calcific AS is projected to more than double by 2050 in developed nations based on simulation models (2). Without definitive management via valvular replacement, the four-year mortality rate is around 45% (3). Over the past decade, transcatheter aortic valvular replacements (TAVR) have exceeded the number of isolated surgical aortic valvular replacements (SAVR) (4). The PARTNER 3 and EVOLUT Low-Risk trials have demonstrated compelling evidence regarding the safety and effectiveness of TAVR compared to SAVR in low-risk patients at 5 and 4 years, respectively (5–8). With the increase in TAVR procedures worldwide across a diverse patient profile with different risk factors, physiological and anatomic constraints challenges have come to the forefront. Traditionally, transfemoral access has shown superior results and is the only TAVR method that showed equal or superior outcomes as compared to SAVR. However, alternative approaches have garnered increasing utility in cases where transfemoral is high risk, or the anatomy is unfavorable. We review the journey, evolution, and the techniques for different approaches of percutaneous TAVR, including transfemoral, transcarotid, transsubclavian/transaxillary, and transcaval approaches, in addition to the traditional “surgical” transaortic and transapical accesses. Consolidating these data highlights each approach's practicality and limitations, thereby providing additional grounding for case-by-case utilization and future clinical research, which will help address significant knowledge gaps.

## Journey and evolution of the transcatheter aortic valve replacement

Historically, patients with severe AS who were considered high risk for surgery were treated either conservatively with medical therapy or by adding an invasive method with balloon aortic valvuloplasty, which was first developed by Dr. Alain Cribier in 1985 (9, 10). Balloon aortic valvuloplasty provided a short-term improvement of valvular function and symptoms and was associated with a high degree of valvular restenosis (11–13). Given the limited therapeutic effect, with substantial risks related to this treatment option, more interest in developing better and more durable solutions with a percutaneous delivery system for a bioprosthetic aortic heart valve was seen. The concept of transcatheter balloon-expandable valves was introduced in the 1980s by Danish researcher Dr. Henning Rud Andersen, who tested the concept with handmade metal stents in pigs (14, 15). Since then, many investigators tested the concept of transcatheter aortic heart valve delivery with prosthetic valves of different designs in animal models (16–20). With that, Dr. Alain Cribier and colleagues performed the first-in-human and proof of concept of the TAVR procedure on April 16, 2002, in Rouen, France (21). That patient had multiple comorbidities and was hemodynamically unstable, which precluded him from obtaining a surgical valve replacement. Due to the large size of the delivery system, a 24F sheath (outer diameter 26F), a venous trans-septal antegrade approach was performed. It was successfully performed with excellent immediate results. Transesophageal echocardiography at nine weeks demonstrated a securely implanted prosthetic valve with continued sustainable function. With that, the TAVR Journey started with Cribier et al. demonstrating the feasibility of safely and successfully implanting a prosthetic heart valve in a native diseased valve using standard interventional techniques (21).

This successful catheter-based approach ushered in a new era of treating aortic stenosis with minimally invasive approaches. The company Criber co-founded, Percutaneous Valve Technologies, was then acquired in 2004 by Edwards Lifesciences Corporation (Irvine, CA, USA), which has continued the development of TAVR valves that are in use today (22).

In 2003, Dr. David Paniagua and his team performed the first human TAVR using the retrograde approach in Texas, USA (23, 24). The Paniagua Heart Valve (PHV) was developed by Endoluminal Technology Research in Miami, Florida. The valve was compatible with 11–16F sheaths. PHV's durability was initially tested *in vitro* on a systemic circulation simulator and multiple animal models, showing proper valve function (23). PHV was mounted on a 16F sheath, allowing a retrograde approach through the femoral artery. The valve immediately functioned normally, improving the patient's clinical status. However, three days post-procedure, the patient died of respiratory failure of unclear etiology (24).

Other pioneer physicians also contributed to the evolution of TAVR technology. In Vancouver, Canada, Dr. John Webb introduced a modified delivery system - a deflectable RetroFlex catheter - which permits easy crossing across the aortic arch and the stenotic valve. It was done with the balloon-expandable Cribier-Edwards Sapien valve retrograde through the femoral artery in 2005 (22, 25, 26). Consequently, Eberhard Grube performed his first human TAVR using another valve design, the self-expanding CoreValve™ device, later acquired by Medtronic, Minneapolis, in 2009 (25, 27).

Subsequently, Friedrich Mohr and Michael Mack evaluated a new alternative approach, the minimally invasive transapical approach, in 2006 in Leipzig, Germany, using the Edwards Ascendra delivery system on 30 patients deemed high risk for surgery (22, 23, 28).

**Figure 1:**
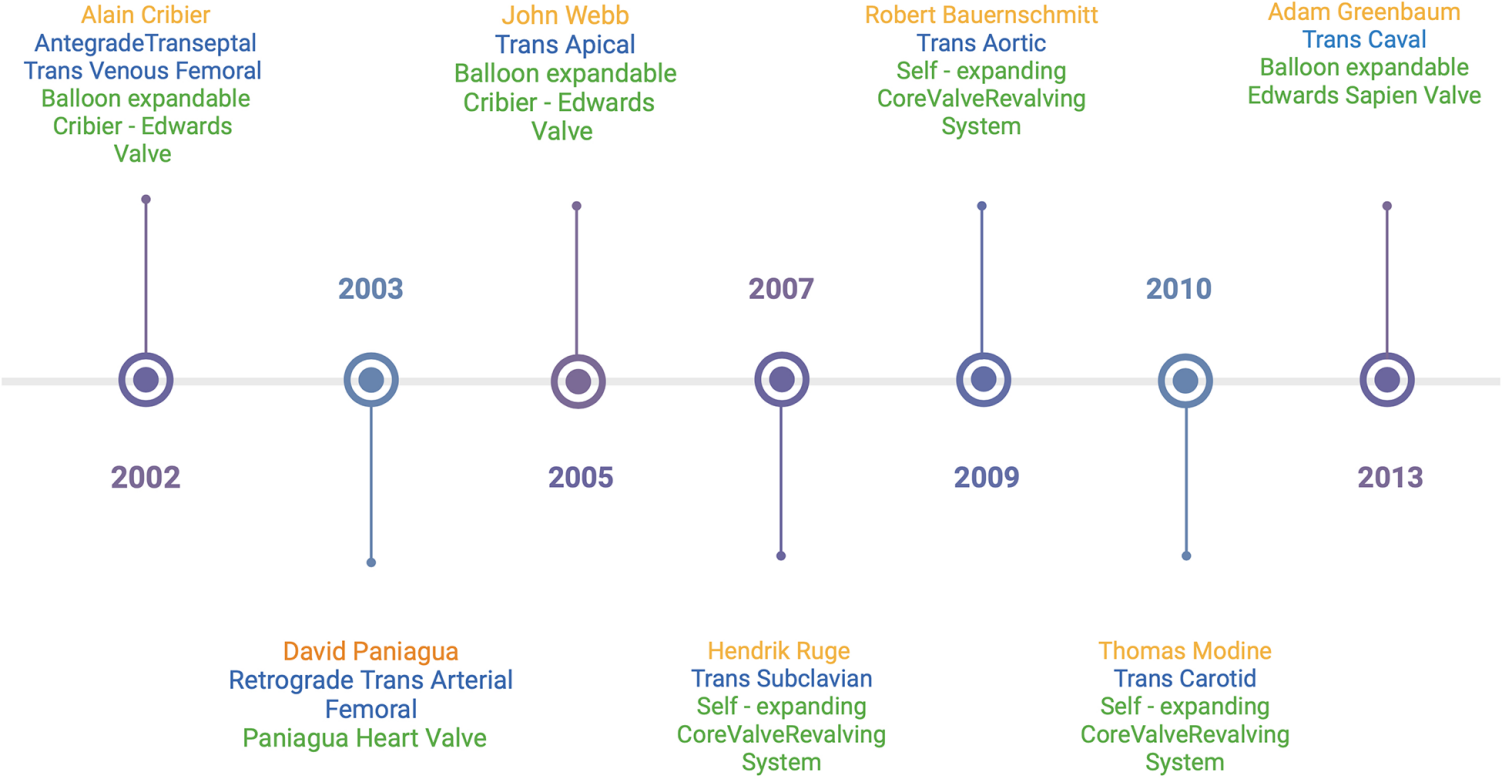
Timeline of first reported cases of TAVR access sites (21, 24, 29–33).

Figure 1 highlights the timeline of the first reported cases of each TAVR access site with their corresponding valve type.

With improvements in TAVR technology seen throughout the years, such as in device size and delivery systems, there have been improvements in clinical outcomes and a reduction in procedural complications (34–36). An improvement in valve size, design, and delivery systems, which are compatible with 14–16 French sheaths, have led to reduced paravalvular leaks and conduction disturbances from valve design and implantation techniques perspective, in addition to lower rates of vascular complications with regards to lower profile deliver systems (35). Different valve designs, such as a self-expanding platform, have improved the flexibility of device delivery and decreased vascular complications due to their compatibility with an Inline sheath. The Inline sheath enables the operator to insert the device without the need for a separate larger access sheath, reducing the overall profile of the system. These diverse valves and systems have distinct features that enable their use in different clinical and anatomical situations (34).

## Access options for transcatheter aortic valve replacement

Transfemoral access has been the most utilized approach for TAVR, accounting for about 95% of cases (4). TAVR access sites can be divided into two categories based on how the arteriotomy is achieved: percutaneous or surgical cut-down. Percutaneous access can be achieved via transfemoral (TF), transsubclavian, or transaxillary (TAx) approaches. Surgical cut-down is typically used for transcarotid (TC), TAx, direct aortic, apical, and, in some instances, transfemoral approaches. A novel percutaneous approach involves transvenous access, with a cross-over to the transaortic, known as transcaval (TCv) access (4).

Surgical cut-down to the femoral artery was a method predominantly used in the first-generation heart valve system. However, with the improvement of vascular closure devices and a decrease in the size of the delivery sheaths, TF TAVR has evolved to percutaneous access. Despite improvement in TAVR technology, challenging access site anatomies such as severely calcified femoral arteries or small femoral artery diameter may sometimes require surgical cut-down with a 4–5 cm skin incision (37). However, this is rarely done anymore. Percutaneous TF access has a higher rate of major vascular complications than TF with surgical cut-down. A study showed that TF with percutaneous access group had a 10% risk of major vascular complications while TF with surgical cut-down had none (37). It may be due to increased vessel wall trauma during sheath exchange. Consequently, surgical access allows the insertion of a sheath under direct field vision, ultimately decreasing injury to the vessel wall. On the contrary, surgical cut-down TF was associated with higher access site infection risk than percutaneous TF, requiring prolonged antibiotics use or wound debridement (38). In addition, surgical cut-down TF access may still warrant the use of general anesthesia.

Preprocedural diagnostic testing often includes cross-sectional imaging and reconstruction of the aortoiliac vessels with multidetector computed tomography (MDCT). An MDCT became the gold standard pre-TAVR test and is required to accurately measure the inner vessel diameters of each femoral, subclavian, and axillary artery. It also enables proper assessment of the aortic root and valve annulus sizing and critical assessment of related structures (39). A multidisciplinary heart team's role is crucial to assessing valvular replacement's suitability, including evaluation of comorbidities, surgical risk, and anatomical characteristics to choose the optimal therapy and access to be used (40).

## Challenges encountered with transfemoral transcatheter aortic valve replacement

Studies have frequently proved the superior clinical outcomes associated with a transfemoral approach for TAVR with respect to other alternative approaches (41, 42). However, in some scenarios, complex vasculature anatomy and conditions have limited the transfemoral approach. Factors such as severe iliofemoral tortuosity, severe vessel wall calcification, inadequate lumen diameter, and significant focal stenosis have been considered challenges, but in some instances, relative contraindications to transfemoral TAVR (39).

Severe iliac tortuosity can be overcome using a bilateral stiff wire technique (39). The success behind this technique is that the bilateral stiff wires promote vessel straightening due to their passive resistance to curvature, although sometimes unsuccessful due to the iliac's high rigidity, making it difficult to straighten them with the bilateral stiff wire technique.

On the other hand, another challenge is severe calcific peripheral artery disease, which can interfere with the ability to deliver prosthetic valves through the femoral vessels (43). Therefore, facilitated TF TAVR can be used, which is TF, following percutaneous interventions using multiple dilators, transluminal angioplasty, iliofemoral stenting, or intravascular lithotripsy (IVL) (44). The use of IVL before attempting transfemoral TAVR has been a topic of interest as a potential method to overcome severe ilio-femoral calcification (45).

Lithotripsy has been used for over 30 years to overcome calcific pathologies such as renal calculi. It delivers pulsatile sonic pressure waves to disrupt calcium deposited in the wall of the vessel (46). Till today, there are no guidelines to specify when it is suitable to perform IVL before TF TAVR. However, Experts have highlighted some CT angiography criteria that enable IVL pre-TF TAVR (46). Kempton et al. stated that if the calcification is localized (less than 20 mm in length) and circumferential (360° calcification circumference), a minimum luminal diameter of 4 mm is required. If the calcification is localized and has a circumference between 270 and 360°, a minimum luminal diameter of 3 mm is needed. If the calcification is diffused (greater than 20 mm in length) and circumferential, a minimum luminal diameter of 4.5 mm is advised. Consequently, if the lesion is diffuse and between 270° and 360° in calcification circumference, a luminal diameter of 3.5 mm is needed (46). A multi-center study was done to assess the safety and efficiency of IVL before transfemoral delivery of a TAVR system in patients with severe aortoiliac calcification. All patients had a successful sheath passage and valve implantation. Access site complications were as low as 4%, with no iliofemoral arterial perforation or dissection seen. Facilitated TF was associated with a lower 30-day rate of major adverse events as compared to trans-thoracic options. Consequently, facilitated TF had a lower 1-year stroke/TIA rate compared to the other extra-thoracic alternative access sites (44). However, with the compelling evidence that highlights the effectiveness and safety of IVL-assisted TF TAVR, complications such as perforation and dissection of the artery can still be seen, which can be life-threatening (47). Multidisciplinary discussion is recommended to assess the best candidates for IVL-assisted TF TAVR through a case-by-case approach.

The rate of IVL use prior to transfemoral TAVR has recently significantly increased. Data from the European TAVR Registry highlighted that IVL before TAVR increased from 2.4% in 2018 to 6.5% in 2020 (48).

## Alternative access sites

The 2019 STS-ACC TVT (Society of Thoracic Surgeons-American College of Cardiology Transcatheter Valve Therapy Registry) Registry report included 276,316 patients undergoing TAVR at sites across the United States. Temporal trends in vascular access sites from the early TAVR period in 2011 till 2019 were analyzed. Femoral access TAVR increased from 57% to 95% in 2019. Consequently, the types of alternative access sites used shifted substantially. Transapical and direct aortic approaches decreased significantly to 0.3% and 0.5%, respectively.

**Figure 2:**
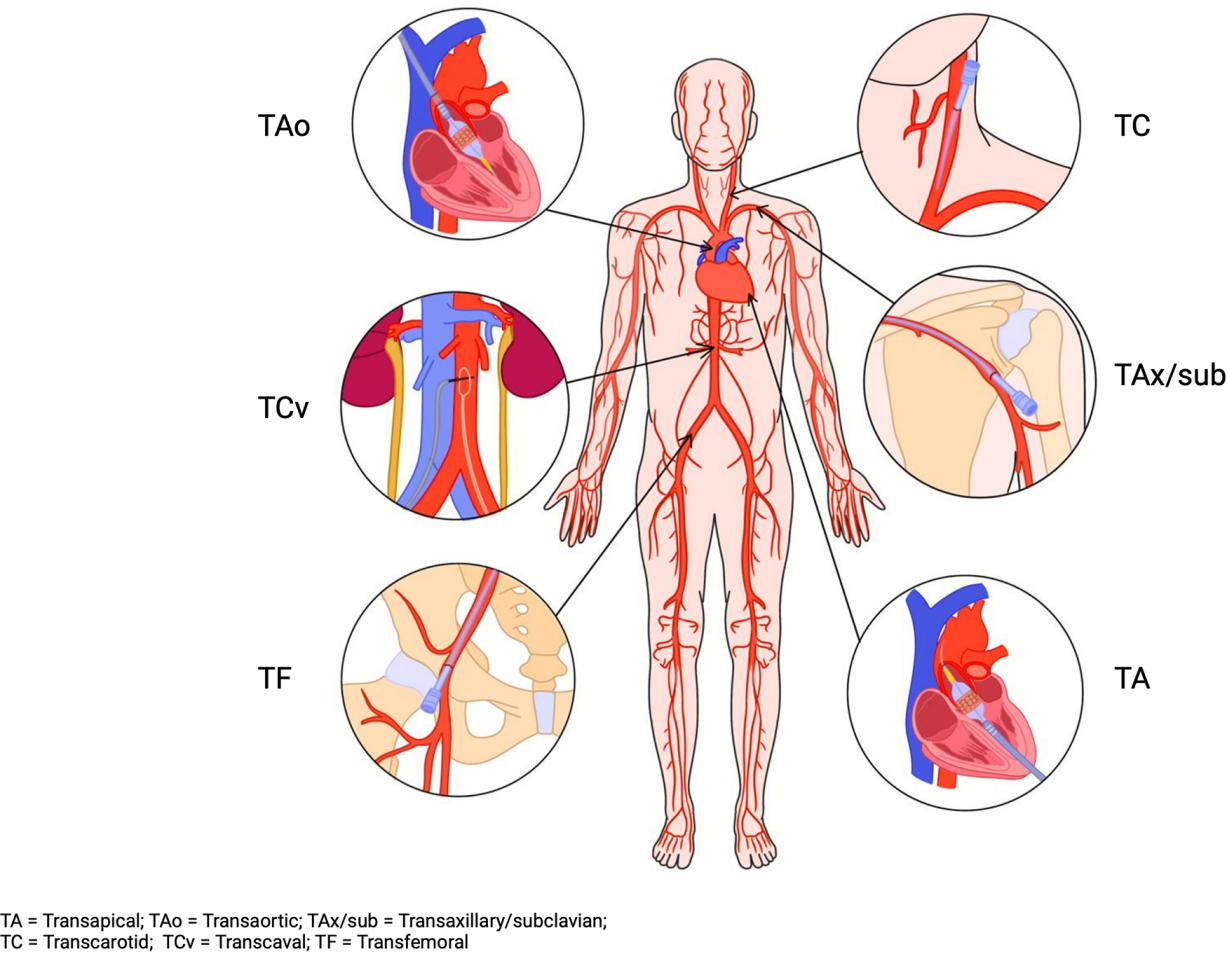
TAVR access sites. TA, transapical; TAo, transaortic; TAx/sub, transaxillary/subclavian; TC, transcarotid; TCv, transcaval; TF, transfemoral.

On the other hand, subclavian access was the most used alternative access site at around 2.5%. Carotid access increased to 0.9%. In addition, transcaval access was added, with 121 cases reported in 2019 (4). The observed temporal trend in TAVR access sites highlights a transformational change in TAVR approaches. Figure 2 highlights the available options for TAVR access sites. The following sections will review the available alternative access sites for TAVR.

## Alternative access sites - transthoracic approach

### Transapical (TA)

TA was first performed in 2005 in Vancouver, Canada (25, 29). The initial impression of this procedure was that it is an off-pump procedure, and large delivery systems can be used regardless of the state of the iliofemoral system or aorta. The operative technique consists of an anterolateral mini-thoracotomy of 5 mm in the fifth intercostal space to access the apex of the heart. The pericardium is incised longitudinally, and the left ventricular apex is punctured. A sheath is then introduced and positioned across the aortic valve, and then the valve is deployed. Then, the transapical sheath is removed with safe suturing of the apex of the ventricle with purse-string sutures to ensure secure hemostasis (49). Contraindications of the TA approach are the presence of left ventricle apical thrombus and severely reduced left ventricular function.

This approach gained recognition at first since it used an antegrade approach of introducing the valve, which was preferred when compared to the first-generation devices of the retrograde transfemoral approach, which were associated with significant peripheral vascular complications, and the other available transseptal femoral venous approach which was associated with procedural complexity and potential injury to the mitral valve. However, TA TAVR lost traction with time due to its high rate of mortality and morbidity, similar to that of surgical aortic valve replacement, which eliminates the benefit of TAVR. The most extensive study to date on TA TAVR showed that the 30-day mortality of TA TAVR was around 8.8%, and the 1-year mortality was 25.6% (50). With the surge of other alternative access sites for TAVR and improvement in the technology of the TAVR valve and their delivery systems, which led to better outcomes, TA TAVR has almost vanished. This is reflected by the 0.3% of TAVR cases reported by the 2019 STS-ACC TVT Registry.

### Transaortic (TAo)

With time, at the TAVR's expense, TAo TAVR rates increased. TAo TAVR was first performed in 2009 in Munich, Germany. It was done in an 80-year-old female patient with critical AS with severe calcification of the iliofemoral system and subclavian arteries (30). TAo can be done through mini sternotomy or mini-thoracotomy. A 6-cm skin incision is done with an extension into the third intercostal space at the level of the innominate artery. Surgeons dissect through the pectoralis muscle, exposing and accessing the ascending aorta through the Seldinger technique. A stiff guidewire is introduced and positioned in the left ventricle to help advance the sheath. Then, the prosthetic valve is deployed in place as per the routine retrograde TAVR technique (51). In order to perform TAo, it is vital to identify a suitable calcium-free target site at the anterolateral ascending aorta with a minimum distance of 5 cm from the aortic annular plane and a convenient trajectory to the landing zone, with the angle of puncture in line with the left ventricular outflow tract (52). TAo has several advantages over TA TAVR, such as the absence of direct myocardial injury and less respiratory compromise while preserving the benefit of having a short distance for the landing zone and better valve manipulation (53). However, the TAo approach has some relative contraindications, such as the presence of a porcelain aorta, short ascending aorta, previous cardiac surgery, and thoracic deformities (52). Supplementary Figure S1 shows access points of the transapical and transaortic TAVR.

TAo TAVR's clinical evidence made it an unattractive TAVR approach. A study showed that the all-cause mortality rate at 30 days of TAo compared to the TF group was 10.9% vs. 4.1% respectively. Major or life-threatening bleeding was significantly higher in the TAo group at 30 days (66.7% vs. 35.4%, *p* < 0.001) (51). In addition, major stroke rate at 30 days was significant for TAo at 5.7% vs. 2.6% for TF. The relatively poor clinical outcomes associated with TAo and TA AVR and the rise in other alternative sites for TAVR with their respective benefits have somehow led to the disappearance of TAo and TA TAVRs.

TAo and TA TAVR are very rarely used and are now mostly mentioned for their historical significance related to the evolution of TAVR therapy.

The following sections will explore contemporary extra-thoracic alternative approaches for TAVR, including transsubclavian/axillary (which can also be accessed through surgical cut-down), transcarotid, and transcaval approaches.

## Alternative access sites - extra-thoracic approach

### Transsubclavian/transaxillary TAVR

Transaxillary access was first reported in 2008 in Munich, Germany (31). Axillary and subclavian access for TAVR has often been combined as one alternative TAVR access site. Most cases in reported registries have described the axillary access (infraclavicular) approach rather than the subclavian approach (supraclavicular) (54). Therefore, throughout the rest of the manuscript, the term transaxillary (TAx) will be used to refer to either transsubclavian or transaxillary. As mentioned, the 2019 STS-ACC TVT Registry reported that TAx was the most utilized site in the alternative access approach for TAVR. An increase in its use as an alternative access site of TAVR was observed, increasing from 20% in 2015 to 49% in 2017 for non-TF-TAVR cases (4).

Atherosclerosis of the iliofemoral system is often more pronounced than that of the axillary or subclavian arteries. In addition, the axillary artery has a vessel diameter similar to that of the iliofemoral artery, around 6 mm. These anatomical characteristics make them reasonable access target sites for TAVR (55).

Pre-procedural screening for TAx TAVR should be done using MDCT, similar to other access types. In patients with chronic kidney disease or other conditions that disallow them to undergo MDCT, arterial duplex ultrasound of the axillary vessels and a non-contrast CT can be done carefully to assess vessel caliber. CT vascular reconstruction of the axillary and subclavian vessels should be done, as typically done for the iliofemoral system. Anatomical characteristics are warranted to perform TAx TAVR, such as the presence of a minimal luminal diameter >5 mm (axillary diameter >5 mm for self-expanding and >5.5 mm for balloon-expandable valves), absence of severely calcified axillary artery, absence of excessive arterial tortuosity, absence of any preexisting vascular injury (i.e., dissection), and presence of an appropriate takeoff angle from the aortic arch (subclavian to arch angulation should be <80°). Consequently, Patients with a known history of coronary artery bypass graft and a patent left internal mammary artery can be considered potential candidates for TAx TAVR if needed (56, 57).

It is essential to identify the segments of the axillary artery during TAx TAVR. Anatomically, the first segment of the axillary artery is the most suitable site for access because, in this area, there are fewer vascular branches than in other areas of the axillary artery (58). Supplementary Figure S2 shows a zoomed-in section of the recommended target zone for the axillary artery access point. Consequently, in this area, the brachial plexus is present as a discrete bundle lying cranial to the axillary artery before dividing into significant branches. Regardless of which axillary segment is accessed, care should be taken to avoid injury to the brachial plexus. On the other hand, if supraclavicular access of the subclavian artery is done, extra caution should be made for the phrenic nerve, which runs superior to the subclavian vessel. Furthermore, the left axillary artery is preferred over the right side. One reason is a better angle for valve delivery in the aortic annulus. In addition, the left-side access site is usually suggested as it closely mimics the transfemoral approach (58).

Axillary artery can be accessed either through surgical cut-down of the artery or percutaneously. For open access, usually, under general anesthesia, operators undergo a sub clavicular skin incision in the deltopectoral groove, followed by subcutaneous tissue dissection through the pectoralis muscle to reveal and isolate the axillary artery. Subsequently, the artery is accessed through the Seldinger technique, a catheter is inserted, and an extra- or super-stiff wire is positioned within the left ventricular apex. A sheath is inserted into the axillary artery, and TAVR follows any regular procedure. At the end of the procedure, the sheath is removed, and the axillary artery is clamped and repaired using interrupted 5-0 Prolene sutures.

On the other hand, for complete percutaneous access, only conscious sedation is needed. The technique used for percutaneous axillary artery access is similar to percutaneous TF TAVR. The artery is punctured with a micropuncture needle under fluoroscopic and ultrasound guidance (57). Proper positioning of the wire in the axillary artery is confirmed under fluoroscopy. The inner cannula of the micropuncture dilator is advanced in the artery. A micropuncture wire is introduced again, and the dilator is upsized. Two Perclose ProGlide devices are placed in the axillary artery for suture-mediated artery closure (57). A sheath is then introduced and placed in the ascendant aorta. After the TAVR procedure, a peripheral balloon is advanced into the left axillary artery for percutaneous access closure and inflated to 1–4 atmospheres at the percutaneous insertion site to allow dry closure. It is done to facilitate proper hemostasis and minimize vascular access complications, but it can also be used in case of residual bleeding to tamponade the vessel. Perclose device is then used for closure (57).

A recent meta-analysis highlighted several observations comparing TAx TAVR to other access sites. The risk of major or life-threatening bleeding was higher via TAx TAVR compared with TF TAVR (OR = 1.51, *p* = 0.034). It might be due to the anatomical location of the subclavian artery, which requires a deep dissection for it to be exposed. In addition, TAx TAVR had a higher 30-day mortality rate (OR = 1.66%, *p* = 0.0002) and all-cause mortality (6–18 month follow-up) (OR = 1.2, *p* = 0.002) than the TF TAVR. The risk of stroke was significantly higher for TAx TAVR compared with TF TAVR and caval approaches (59). Consequently, a cohort study using the Society of Thoracic Surgeons/American College of Cardiology TVT Registry which included 1249 patients who underwent TAVR using TAx access showed a significantly higher stroke rate at 6.3% (54). This elevated stroke rate is consistent with the CoreValve Extreme Risk US Pivotal Trial data. However, the stroke rate was much higher than recorded in the PARTNER 2 trial. This discrepancy in stroke rates among several studies can be due to factors that need further investigation. Several questions should be asked, such as whether there is a benefit to using cerebral embolic protection devices during TAx access or whether there is a relationship between higher stroke rates and the side of the axillary access site used.

### Transcarotid TAVR

The transcarotid approach was first described in 2010 in France in a patient with severe tortuosity of the iliofemoral and subclavian arteries (32). It was then considered as a last resort due to the theoretical association of increased stroke risk from embolic events arising from carotid atheroma and valvular calcification.

Routine preoperative evaluations are needed, including carotid artery ultrasonography and computed tomography angiography of the chest, abdomen, and pelvis. Carotid site selection is determined according to several criteria, including minimal lumen diameter, degree of tortuosity, calcification, and percentage of contralateral carotid artery stenosis. A lumen diameter of 6.5 mm is considered the minimum accepted diameter for TC TAVR (60). Absence of vertebral, subclavian, and contralateral common carotid stenosis and congenital variants of the aortic arch is also recommended. If there is stenosis of more than 50% at the selected common carotid artery, this warrants using this side as the preferred access one to maximize cerebral flow from the contralateral patent artery. Some experts recommend the evaluation of patients who might be prone to cerebral hypoperfusion using cerebral magnetic resonance angiography with transcranial Doppler ultrasound to evaluate the circle of Willis and collateral cerebral blood flow. Intraoperatively, monitoring cerebral perfusion through cerebral oximetry or electroencephalography might be used to evaluate the need for carotid shunting (61). However, these perioperative neurovascular workups are not routinely done. Until today, no general consensus exists on using the previously mentioned diagnostic tools to accurately stratify candidates' risks of the TC TAVR procedure and its neurological burden.

This so-called “minimally-invasive nature of the procedure” is done through a cut-down approach with a 4 cm incision medial to the sternocleidomastoid muscle just above the clavicle to expose the common carotid artery. Care should be taken when dissecting the carotid sheath due to the potential risk of injuring nearby neurovascular structures, notably the vagus nerve. The carotid artery is accessed, and the sheath is placed using the Seldinger technique (60). Supplementary Figure S3 highlights steps in obtaining access to common carotid artery for TAVR. The TAVR sheath is positioned in place, and the valve is deployed. Upon the removal of the sheath, the common carotid artery is clamped proximally and distally, and the arteriotomy is repaired.

Allen et al. highlighted an important observation regarding the stroke risk and TC TAVR technique (62). The initial stroke risk with TC access in the French Transcarotid TAVR registry, which included 96 patients, was 6.3% (63). The French technique used by Mylotte and his colleagues included sequentially dilating the carotid artery before introducing the delivery sheath without clamping the distal portion of the common carotid artery during the procedure. However, the contemporary technique used for TC access evolved and includes distally clamping the common carotid artery during the procedure and using a transverse arteriotomy without serial dilation of the artery. After valve delivery, the artery is flushed for potential embolic remnants and repaired before the carotid clamp is released. This technique was described as a single-center experience report in two different institutions and was associated with stroke rates as low as 0%–2.4% (60, 64). Furthermore, an updated report from the French Transcarotid TAVR registry showed a significantly lower stroke rate of 1.6% compared to their initial report of 6.3%. This can be attributed to the TC access technique, which included transverse carotid arteriotomy with carotid clamping (65). Therefore, it is essential to highlight the observed association between the TC access technique and stroke rate.

Several studies have tried to evaluate the safety and outcomes of the transcarotid approach as an alternative access site for TAVR. Supplementary Figure S4 summarizes the results of a meta-analysis by Usman et al., which displays the incidence of different outcomes in TC TAVR (66). The study also highlighted an important observation where the incidence of stroke and TIA decreased throughout the years of performing TC TAVR which might be attributed to improved skill acquisition and operator experience. Furthermore, a lower major vascular complication was seen (pooled estimate 2.4%) when compared with the PARTNER 2 trial (7.9%) (67) and SURTAVI trial (6%) (33). A recent meta-analysis compared TC TAVR data against each alternative access site. The key findings were as follows: TC had higher short-term mortality rates and lower rates of vascular complications than transfemoral. TC was associated with a lower mortality rate than that of transaortic and transapical access. Compared with transaxillary access, TC had lower contrast volume, faster procedure time, and lower rate of major vascular complications. However, TC was associated with higher major bleeding than transaxillary access. More importantly, when it comes to TC access, no significant increase in CVA events was seen compared to other accesses (68).

With the increase in operator experience with TC TAVR and the compelling evidence of its safety and effectiveness, TC TAVR has increased compared to other alternative accesses. Some centers have utilized TC access as the preferred alternative access site for TAVR (60).

### Transcaval TAVR

**Figure 3:**
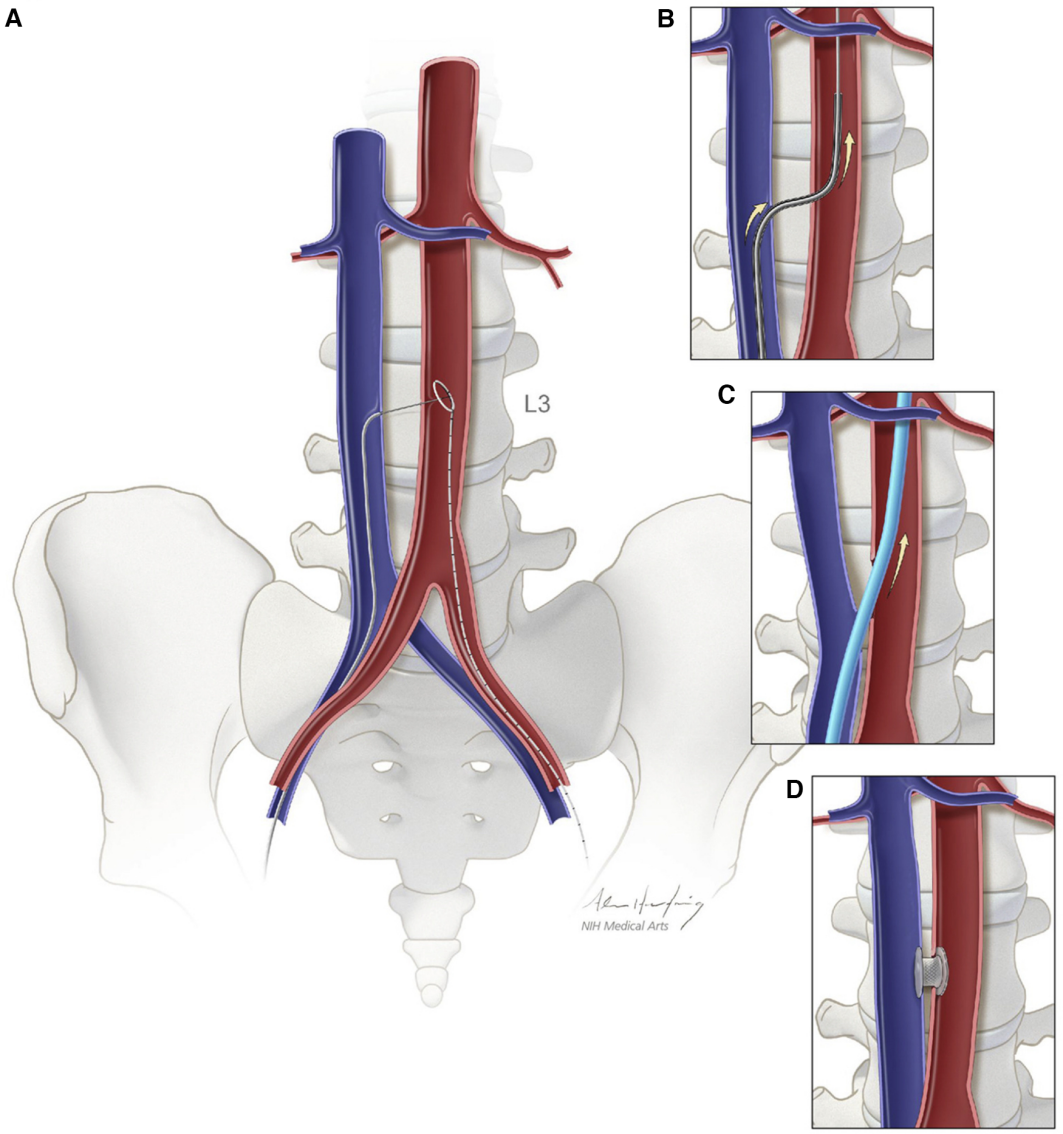
Schematic depiction of caval-aortic access. (**A**) A catherter directs a transfemoral vein guidewire from the inferior vena cava towards a snare target positioned in the adjoining abdominal aorta. (**B**) A catheter is advanced over the guidewire into the aorta and used to introduce a more rigid guidewire. (**C**) The valve introducer sheath is advanced from the vena cava into the aorta. (**D**) After completion of TAVR, the aorto-caval access tract is closed with a nitinol occluder. Reprinted from Greenbaum AB, O'Neill WW, Paone G, Guerrero ME, Wyman JF, Cooper RL, Lederman RJ. Caval-aortic access allow transcatheter aortic valve replacement in otherwise ineligible patients: initial human experience. J AM Coll Cardiol. 2014 Jul 1:63., with permission from Elsevier.

Transcaval (TCv) access initially originated as a concept in 2010 as an alternate method to deliver larger caliber delivery devices to the abdominal aorta from extra-thoracic access. With additional testing in animal models, the first-in-human TCv TAVR procedure was performed in 2013 (69, 70). Since then, in addition to TAVR, TCv access use has expanded to help deliver transcatheter endovascular aortic repair (TEVAR), transcatheter temporary mechanical circulatory support (tMCS) when limb ischemia is of concern, and in pediatric interventional cardiology. TCv TAVR involves the placement of an introducer sheath into the femoral vein with the advancement of an electrosurgical guidewire into the inferior vena cava (IVC). Pre-procedural MDCT-based evaluation of a calcium-free target window in the infrarenal abdominal aorta is localized with fluoroscopy. The electrosurgical wire is then used to traverse the wall of the IVC, through the retroperitoneal space, and into the aorta at the planned aortotomy site, thereby creating an arteriovenous fistula. The electrosurgical wire is then snared and exchanged for a rigid wire, and the procedure proceeds via a standard approach for retrograde TAVR. Post-delivery, the TCv tract is closed with a nitinol occluder device, and a closure aortogram is completed to identify any complications. Often, if existent, minimal residual aorto-caval fistula is acceptable. However, severe extravasation may require covered stenting or rarely surgical repair (69). Figure 3 highlights a schematic depiction of caval-aortic access as described above.

The TCv approach utilizes the physiology of the retroperitoneal space to its advantage. In prior observation surgical experience, it was often thought that aortic perforation in the retroperitoneum would cause life-threatening hemorrhage. However, this is only true for an open retroperitoneum, as the hydrostatic pressure of the space is compromised. In situ, the retroperitoneal hydrostatic pressure is around 20 mmHg, higher than that of the IVC, around ten mmHg. Thus, any aortic blood extravasation will favor the IVC instead of the retroperitoneum, creating a well-tolerated shunt without significant hemodynamic consequences (71, 72).

Transcaval target selection is primarily based on sufficiently large non-circumferential calcium-free aortic window (typically >2 mm larger than delivery sheath), absence of interposed tissue such as bowel or mesenteric arteries, and adequate distance from aortic branches in case there is a need for covered stent bailout. Non-ideal targets include those with synthetic grafted endovascular repair or aortic aneurysm; however, these are still suitable. Relatively contraindicated targets include aortic dissection, pedunculated atheromata, or aortic stents (72). IVC filters are considered soft contraindications because TCv can be done above, below, or alongside the filter. It is recommended to identify bony landmarks like the lumbar spines and the iliac crest on CT scan and angiographic landmarks (aortoiliac bifurcation and renal artery) by fluoroscopy to ensure safe TCv access and closure. These images can be displayed during the procedure to help guide the operator.

In current practice, the aorto-caval tract is closed using the off-label nitinol cardiac occluder device - Amplatzer Duct Occluder. This device demonstrated favorable and acceptable results (72). To be noted, this occluder is incompletely hemostatic directly after deployment, prone to pull-through, and may result in the need for blood transfusion or covered stent usage (73). In addition, some patients with significantly impaired right ventricular function may poorly tolerate persistent left-right shunting and should be closely monitored. A first-in-human test of a dedicated Transcaval Closure Device was developed and studied for 12 patients undergoing TCv. The study showed promising outcomes, where all patients had complete closure of the transcaval tract 30 days post-discharge and the absence of vascular or bleeding complications (73). However, further trials with a larger sample size are needed if widespread adoption of this approach will take place.

In clinical outcomes studies, transcaval access is a safe approach compared to other alternative access techniques. Barbash et al. investigated 185 patients, of whom 12% underwent transcaval TAVR, and 82% underwent alternative access TAVR. Their results noted a lower incidence of AKI in the transcaval cohort and shorter hospital stays but no difference in early or 30-day mortality (74). Lederman et al. conducted a meta-analysis of transcaval access with transaxillary access and concluded lower stroke and similar bleeding rates across eight centers in the United States (75). A recent systematic review comparing TCv TAVR against supra-aortic (TC and TAx) TAVR found no statistically significant difference between in-hospital or 30-day all-cause mortality, major bleeding, need for blood transfusions, major vascular complications, and kidney injury. Notably, TCv TAVR was associated with a lower rate of neurovascular complications but failed to reach statistical significance (76).

## Discussion

With increasing technological advancements in TAVR devices and delivery systems, in addition to growing operator experience, the number of TF TAVR procedures across a diverse patient profile with different risk factors continues to increase, becoming the vast majority of daily cases performed. Alternative access site approaches have been an essential and needed solution for the minority of patients who cannot undergo TAVR through the femoral approach. Facilitated TF TAVR, with the use of multiple dilators and/or IVL-assisted TF access, has somehow limited the need for alternative access sites for patients previously considered unable to undergo TF TAVR. It has demonstrated promising outcomes (44, 45, 47, 77). However, randomized studies on its use are still lacking. When alternative access is needed, current evidence strongly recommends an extra-thoracic approach rather than a thoracic one, which limits the role of both TAo and TA TAVR (Graphical Abstract) (50, 51, 53). TAx and TC approaches have been the most utilized alternative approaches (4). Many centers have depended on the TC approach as their first alternative access site (60). The TC approach's strong evidence on stroke risks has made it a compelling alternative for patients with unfavorable anatomy for TF TAVR (60, 64–66). On the other hand, TCv access has demonstrated favorable outcomes (74–76). However, some limitations remain that should be addressed, such as its significant learning curve, available data regarding its bleeding risk, effect of the potential remaining large vessel shunt, availability of specialized materials needed, and its relatively higher cost. TCv is currently considered to be an off-label treatment option for TAVR. Tables 1, 2 highlight the major studies on outcomes for alternative TAVR access methods and their procedural characteristic, mainly operator position and anesthesia mode, respectively (52, 57, 74, 83, 84).

**Table 1:** Summary of Major studies on outcomes for alternative TAVR access methods.

Method	Studies	Study period	Patients	Major/Life threatening bleeding	Major vascular complications	Stroke	MI	30-day mortality
TA	Thourani et al. (50)	2011–2014	4,085	N/A	14 (0.3)	86 (2.1)	37 (0.9)	359 (8.8)
	Frohlich et al. (78)	2007–2012	761	N/A	3 (0.4)	23 (3)	N/A	80 (11)
TAo	Thourani et al. (50)	2011–2014	868	N/A	3 (0.3)	22 (2.5)	3 (0.3)	89 (10.3)
	Frohlich et al. (78)	2007–2012	185	N/A	6 (3)	1 (1)	N/A	14 (7.6)
TC	Beurtheret et al. (43)	2013–2017	911	91 (9.99)	2 (0.22)	33 (3.62)	3 (0.33)	34 (3.73)^a^
	Kirker et al. (79)	2015–2019	788	1 (0.1)	12 (1.5)	32 (4.2)	N/A	32 (4.3)
	Folliguet et al. (80)	2013–2015	435	40 (9.2)	14 (3.2)	19 (4.4)	3 (0.7)	15 (3.4)
	Debry et al. (81)	2010–2018	201	11 (5.7)	17 (8.5)	14 (6.8)	N/A	9 (4.5)
TSc	Kirker et al. (79)	2015–2019	1,576	2 (0.1)	35 (2.2)	114 (7.4)	N/A	80 (5.2)
	Beurtheret et al. (43)	2013–2017	702	47 (6.7)	9 (1.28)	21 (2.99)	1 (1.14)	30 (4.27)^a^
	Frohlich et al. (80)	2007–2012	188	N/A	4 (2)	9 (5)	N/A	5 (2.9)
	Debry et al. (81)	2010–2018	113	4 (3.6)	10 (9)	4 (3.2)	N/A	6 (5.5)
TCv	Lederman et al. (75)	2017–2020	238	24 (10.1)	6 (2.5)	6 (2.5)	N/A	14 (5.9)
	Paone et al. (82)	2015–2017	58	10 (17.2)^b^	1 (1.7)	1 (1.7)	N/A	2 (3.5)

^a^ Procedural mortality (In-hospital or 30-days mortality).

^b^ Reported as retroperitoneal bleeding.

**Table 2:** Summary of procedural characteristics of different TAVR access methods- operator position and anesthesia mode (52, 57, 74, 83, 84).

Anatomic regionality	Groin	Neck/Upper chest	Mid chest
Access modality	Transfemoral	Transcarotid	Transapical
	Transcaval	Transsubclavian/Transaxillay	Transaortic
Anesthesia type	MAC or Moderate sedation >> General	General >> MAC > Moderate sedation	General
Primary operator positioning	Right hip	Ipsilateral to access, at the head of the table	Left upper chest
Secondary operator positioning	Right knee	Ipsilateral to access, shoulder or upper arm	Left mid chest or Right upper chest (across from primary operator)
Anesthesia positioning	Head of table	Contralateral to access, at the head of the table	Head of table

MAC, monitored anesthesia care.

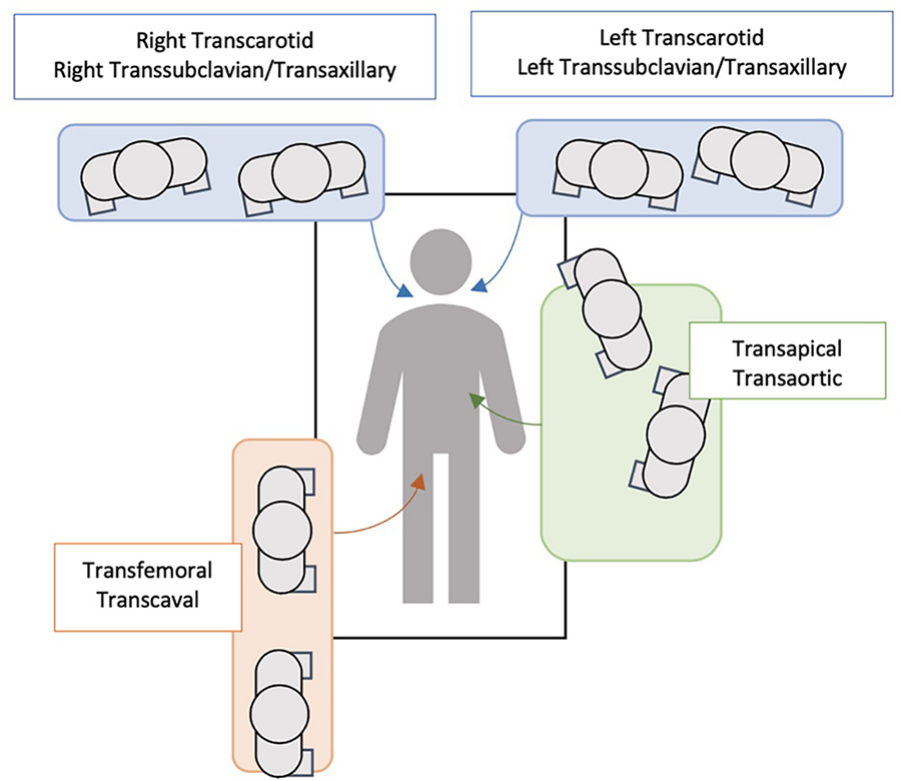

Only observational studies have compared the different alternative approaches. Therefore, randomized controlled trials are still warranted to safely ensure an optimal approach for choosing the appropriate access site for a given patient profile. However, conducting such studies would be complex since this alternative approach comes as a second choice when the transfemoral approach is not an option, which would limit inclusion (61). In addition, anatomical conditions that enable an alternative approach over the other would promote selection bias in a comparative study. Subsequently, institutional expertise in one approach over another would yield inconsistent results.

Notably, a multidisciplinary heart team consisting of imaging experts, interventionalists, and cardiac surgeons should be the cornerstone of any TAVR center of excellence. Proper planning of TAVR procedures yields superior outcomes. Expert consensus regarding operator and institutional recommendations for TAVR has emphasized the importance of a TAVR center to expand to at least one alternative access method other than TF TAVR (85). With the available data, no one extra-thoracic alternative approach for TF TAVR is preferred over the other. Each approach has its advantages and risks, which should be assessed on a case-by-case basis. Every TAVR center should have an algorithm for the TAVR access site strategy. This depends on the TAVR volume, expertise, and personal preference of the operators with each access site. Therefore, TAVR centers can rely on their team's expertise to safely choose the best alternative access site in each case, based on available data, personal preference, and skills set.

**Figure 4:**
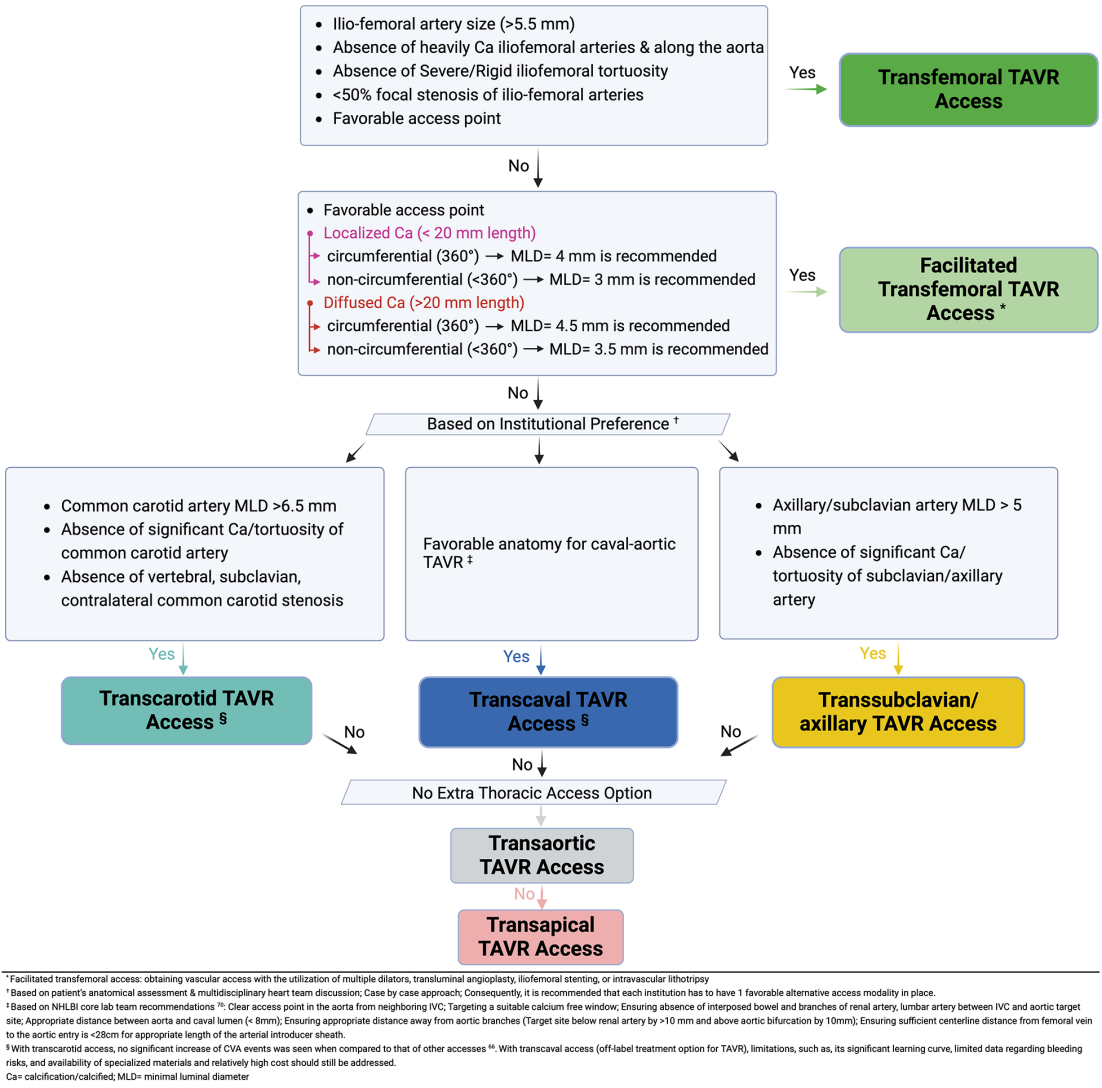
Multidisciplinary structural heart team algorithm for TAVR access site suitability (46, 70).

Figure 4 represents an algorithm solely based on the authors' personal opinions on what they use in their practice, highlighting the approach used to choose TAVR access sites.

Interventionalists continue to explore TAVR through other alternative access sites. Recently published reports describing attempted TAVR through the brachial artery had promising early results (86–88). Although access through the brachial artery may present as an attractive option, multiple limitations and risks are associated with this approach (86, 87). On the other hand, biotechnology research and development innovators are constantly working on novel TAVR delivery systems, devices, and leaflet technologies to safely expand TF TAVR to almost all patients, regardless of their risk profile, which may ultimately limit alternative access approaches even more (34).

## Conclusion

Alternative approaches for transfemoral TAVR currently remain an essential option in a subset population with unfavorable iliofemoral vasculature anatomy. The ongoing development of future pipeline technologies in TAVR valves and delivery systems may help minimize the current limitations and refine procedural techniques. Future studies on upcoming novel TAVR devices and delivery systems would be interesting to see how they will affect our current alternative approaches for TAVR.
