# Supplementary figures and images for: Alternative access in transcatheter aortic valve replacement—an updated focused review
Source: Front Cardiovasc Med. 2024 Aug 8;11:1437626. doi: 10.3389/fcvm.2024.1437626 (PMC11338806; doi:10.3389/fcvm.2024.1437626)

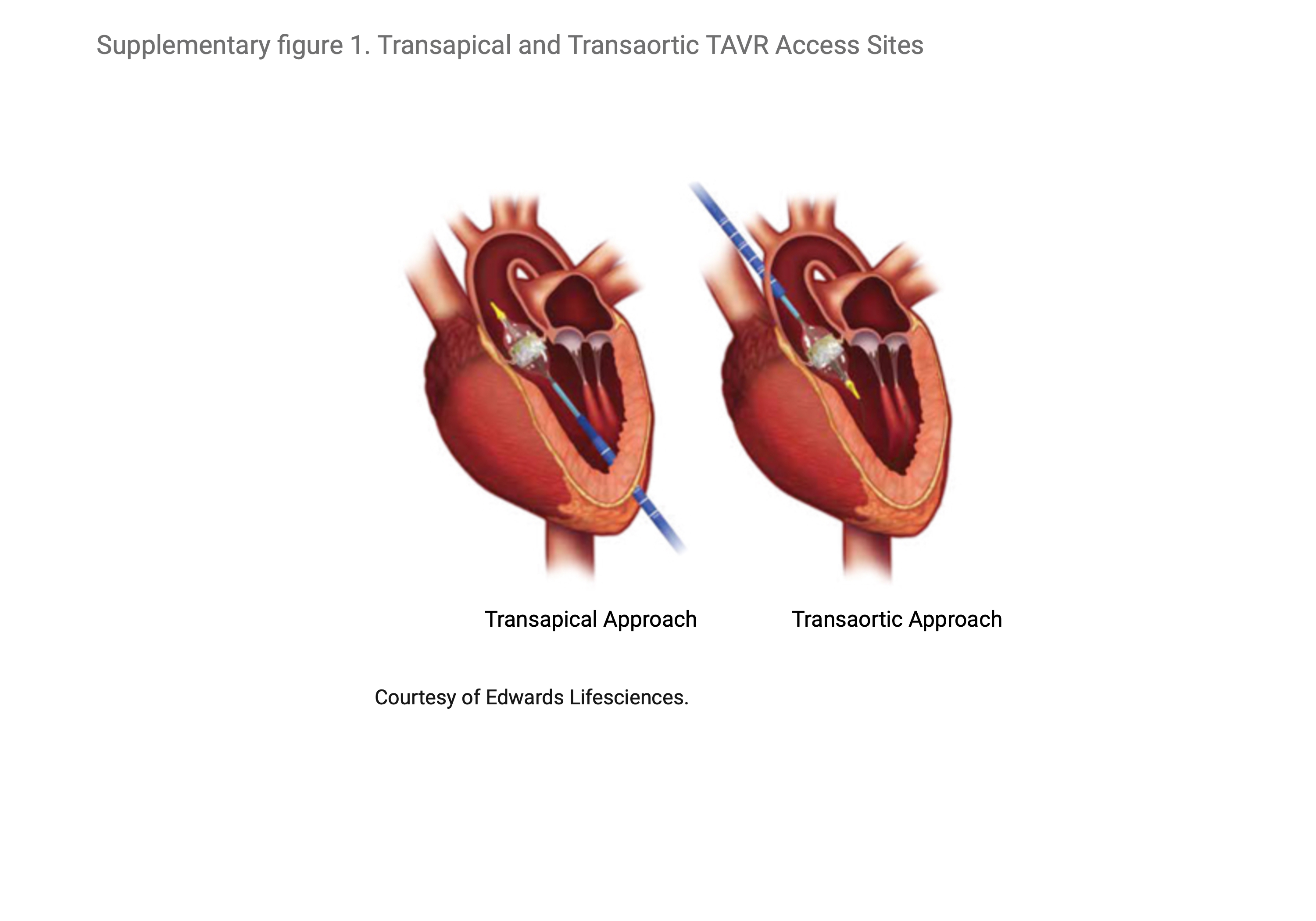

Supplement: Supplementary file 1 [file Image1.jpeg]

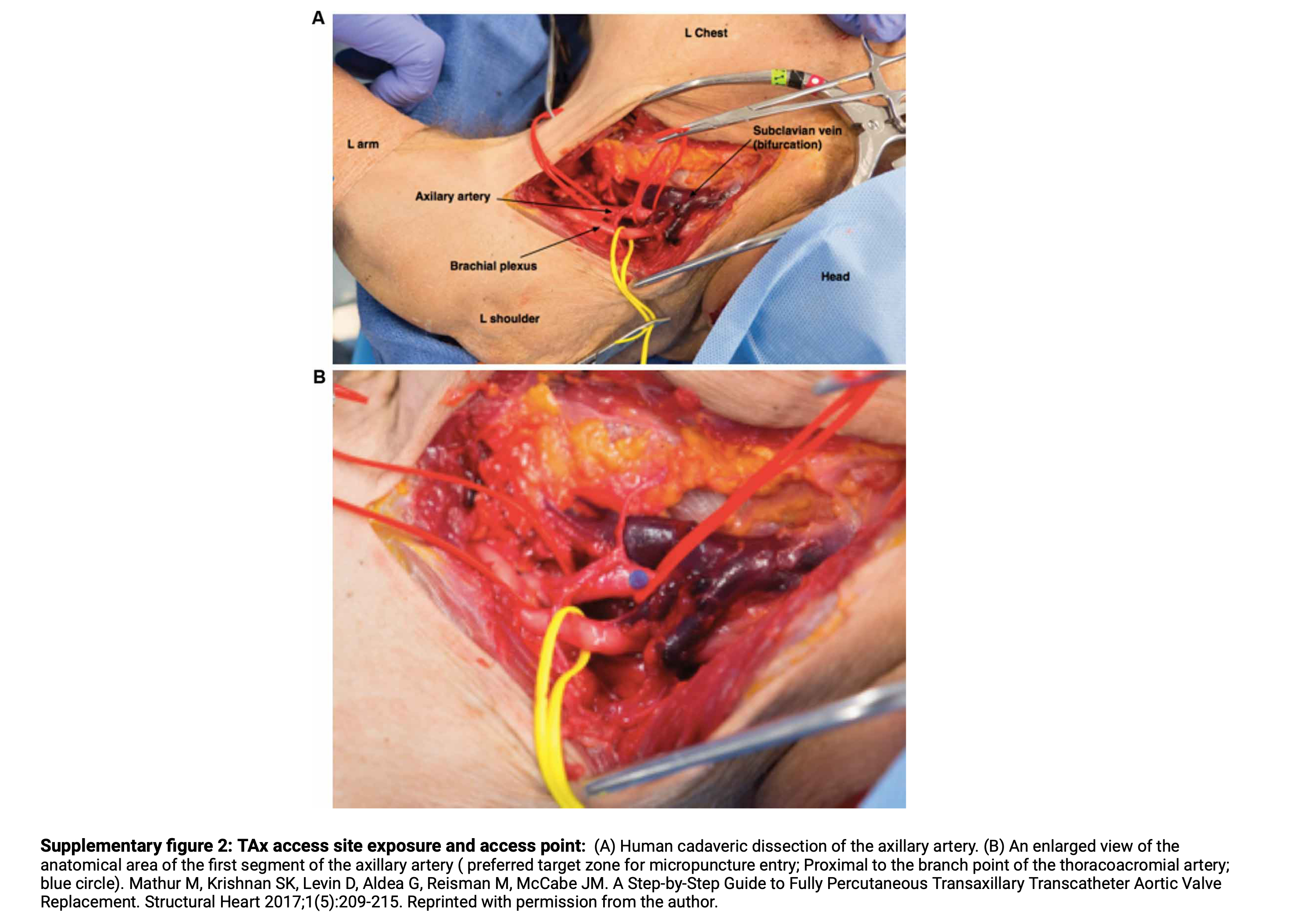

Supplement: Supplementary file 2 [file Image2.jpeg]

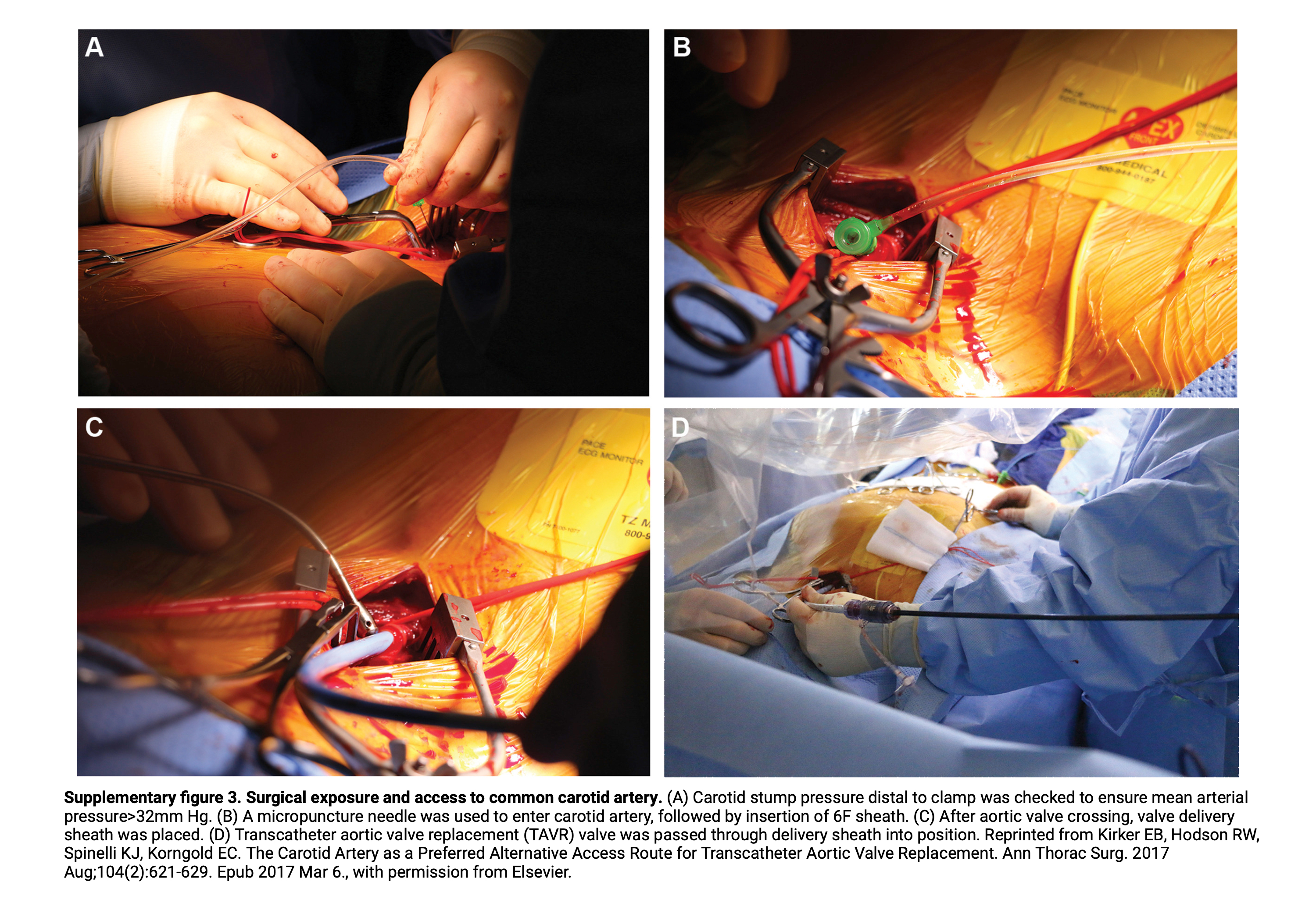

Supplement: Supplementary file 3 [file Image3.jpeg]

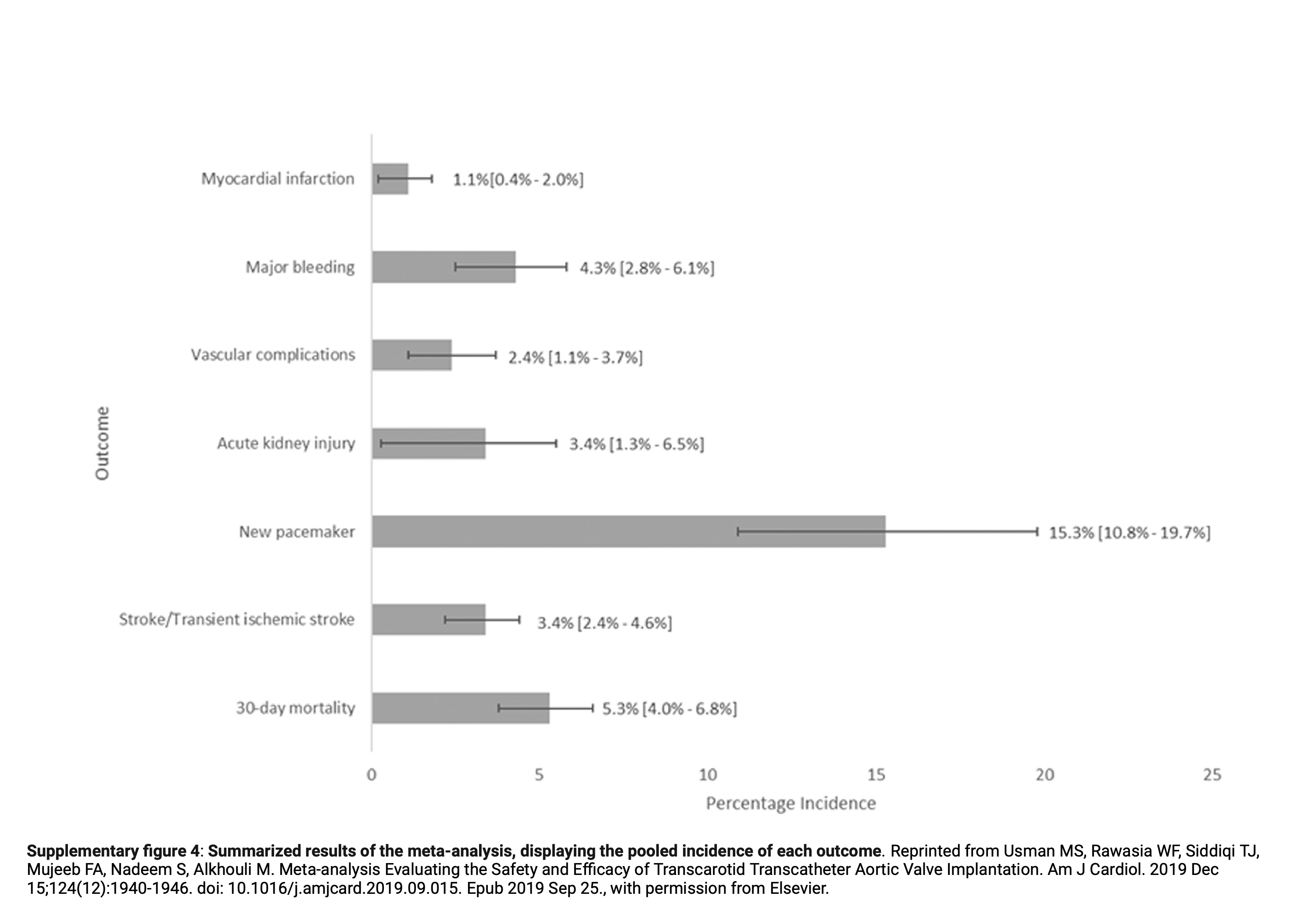

Supplement: Supplementary file 4 [file Image4.jpeg]
